# Antimicrobial stewardship in Latin America: Past, present, and future

**DOI:** 10.1017/ash.2022.47

**Published:** 2022-04-22

**Authors:** Valeria Fabre, Sara E. Cosgrove, Clara Secaira, Juan Carlos Tapia Torrez, Fernanda C. Lessa, Twisha S. Patel, Rodolfo Quiros

**Affiliations:** 1 Division of Infectious Diseases, Department of Medicine, Johns Hopkins University School of Medicine, Baltimore, Maryland, United States; 2 Universidad del Valle, Guatemala City, Guatemala; 3 Clínica Ángel Foianini, Santa Cruz de la Sierra, Bolivia; 4 International Infection Control Program, Division of Healthcare Quality Promotion, National Center for Emerging and Zoonotic Infectious Diseases, Centers for Disease Control and Prevention, Atlanta, Georgia, United States; 5 PROAnet Project Lead, Buenos Aires, Argentina

## Abstract

Implementation of antimicrobial stewardship programs (ASPs) in well-resourced countries has been associated with reductions in antibiotic-resistant infections and improved patient outcomes. Several guidance documents providing recommendations on how to structure antimicrobial stewardship activities at the national and hospital level in resource-limited settings have been published. However, few hospitals in Latin America report having a structure or resources needed for a successful ASP. Given the alarming increases in antimicrobial resistance in Latin America, better understanding of barriers to promote implementation of effective ASPs is urgently needed. We have summarized past and present antimicrobial stewardship activities in Latin American hospitals, and we describe key elements needed in future efforts to strengthen antimicrobial stewardship in the region.

## Background

Antimicrobial resistance (AMR) in Latin America has reached critical levels. Carbapenem nonsusceptibility among gram-negative organisms has increased drastically in the region from 0.3% in 2002 to 21% in 2016, with some countries reporting a prevalence of 20%–50% according to the Latin American Network for Antimicrobial Resistance Surveillance (also known as ReLAVRA, its Spanish acronym).^1^ Several South American countries are reporting the emergence of new carbapenem-resistant Enterobacterales (CRE) that produce >1 carbapenemase.^2^ These trends highlight the urgent need to build and strengthen antimicrobial stewardship activities in the region. National plans including both antimicrobial stewardship and infection prevention and control (IPC) programs can result in significant reductions in the spread and incidence of multidrug-resistant (MDR) organisms, including CRE.^3^

According to the Pan American Health Organization (PAHO), 30 of 33 countries in Latin America and the Caribbean are in the process of developing or have completed national action plans to combat AMR, and 19 of 33 countries report to the Latin American Network for Antimicrobial Resistance Surveillance.^4^ We have summarized the history of hospital antimicrobial stewardship activities in Latin America. We have described current hospital antimicrobial stewardship activities, and we have highlighted unaddressed issues and gaps to effective implementation of ASPs in the region. We use the term Latin America to refer to countries south of the United States, not including the Caribbean.

## Evolution of antimicrobial stewardship activities in Latin America

In 1997, the Infectious Diseases Society of America and Society for Healthcare Epidemiology of America published Guidelines for the Prevention of AMR that included both strategic goals (eg, tracking antibiotic use) and general approaches to improve antibiotic use in hospitals (eg, antimicrobial restriction).^5^ At the time, however, data were limited regarding both the effectiveness of these approaches and how to accomplish strategic goals. In 1994, Quiros et al^6^ showed a significant reduction in antimicrobial use (12% overall reduction measured in defined daily dose; *P* < .01) and in inappropriate prescribing (21% based on point-prevalence surveys; *P* < .05) with implementation of hospital-specific treatment guidelines, preauthorization, autostop of antibiotics at 24 hours for surgical prophylaxis, and daily handshake stewardship at a tertiary-care center in Argentina. Similarly, in 1999, Bantar et al^7^ showed that implementation of an antimicrobial stewardship program (ASP) including implementation of a prescription form, education, and prescribing control at a 250-bed public tertiary-care center in Argentina resulted in a significant improvement in antibiotic use as well as methicillin-resistant *Staphylococcus aureus* (MRSA) and carbapenem-resistant *P. aeruginosa* infections, crude mortality, and length of stay. The intervention was developed and implemented by a multidisciplinary team including an infectious disease (ID) physician, a clinical microbiologist, 2 pharmacists, and 1 data analyst. This team had the critical advantage of having resources to extract and analyze data, and they integrated key disciplines in the management of antibiotics such as microbiology, medicine, and pharmacology.

Despite these early successful demonstrations, many hospitals in Latin America have struggled to implement multidisciplinary teams to execute effective antimicrobial stewardship activities. A 2015 survey assessing the development of ASPs in 27 hospitals in 10 countries throughout Central and South America showed that 59% of hospitals had a written statement supporting antimicrobial stewardship activities.^8^ However, 63% lacked a pharmacist dedicated to antimicrobial stewardship activities, 33% did not have treatment guidelines (based on either international or national recommendations or guided by local susceptibility patterns), and only 48% performed prospective audit and feedback.^8^ Although most participants (78%) reported developing periodic antibiograms, only half of them shared the information with their prescribers. Although 48% reported access to information and technology support for antimicrobial stewardship activities, only 44% monitored antibiotic use. These data may be an overestimate of the current situation of ASPs in the Latin America region because hospitals with better established ASPs may have been more likely to participate in the study. Moreover, an earlier global survey on antimicrobial stewardship also showed low ASP implementation in Central and South America: only 46% of Central and South American hospitals had implemented an ASP.^9^

In 2016, Quiros et al^10^ led a study evaluating implementation of ASPs in 111 hospitals in Argentina through a scored self-assessment based on Centers for Disease Control and Prevention (CDC) core elements (ie, leadership support, antimicrobial stewardship interventions, monitoring of antibiotic use and AMR, and education). These investigators found that, on a scale from 0 to 100 (a higher score indicating a more advanced program), only 28 (25%) of 111 participating hospitals had an average score of 52, whereas 75% scored an average of 32. Most sites scored poorly in training and education, and having a full-time ID physician was associated with a higher score. More recently, the impact of ASPs in 77 intensive care units (ICUs) from 9 Latin American countries (Argentina, Bolivia, Brazil, Chile, Colombia, Ecuador, Panama, Peru and Uruguay) was evaluated through a similar self-assessment and point-prevalence surveys.^11^ In this study, hospitals with a high global score on the self-assessment (ie, more robust programs) had lower antibiotic consumption (both lower defined daily doses and fewer antimicrobials per patient), lower rates of hospital-acquired infections due to MDR organisms, and were more likely to have more robust IPC programs than hospitals with a low score. Like the previous study, education, tracking antibiotic use, and assessment of antibiotic appropriateness scored lowest (ie, not carried out or partially implemented).

Lastly, several studies have shown that ASPs are cost-effective in Latin America.^7,12,13^ The previously mentioned study by Bantar et al^7^ showed that implementation of the ASP resulted in US$1,000,000 in cost savings. One study evaluating the cost-effectiveness of 2 antimicrobial stewardship strategies in a 550-bed university hospital in southern Brazil found that while more labor intensive, an intervention that included prospective audit and feedback through face-to-face interactions was more cost-effective than the intervention that included review of antimicrobials by the clinical pharmacist and discussion of recommendations with the ID physician only (US$19,317 incremental cost-effectiveness ratio, representing the cost per incremental patient that survives 30 days),^12^ underscoring the value of interacting with prescribers.

## Gaps in antimicrobial stewardship implementation

A recent scoping review evaluating the implementation of hospital ASPs in Latin America and the Caribbean showed that although there has been a steady increase in published studies on antimicrobial stewardship interventions in recent years, ∼60% of the reports come from only 3 countries (Brazil, Argentina, and Colombia), suggesting differences in antimicrobial stewardship stage development among countries in the region.^14^ Next, we summarize key elements to promote effective antimicrobial stewardship in Latin American hospitals.

### Underfunded public health and hospital infrastructure

Although Latin American countries share the origin of the languages spoken (those derived from Latin such as Spanish, French, and Portuguese) they have very diverse sociopolitical environments, economies, and health systems^15^ that affect implementation of effective measures to counteract AMR. In many Latin American countries, limited national resources are dedicated to public health, including AMR; thus, infrastructure to allow for tracking AMR and antibiotic use rates at the national level are scarce.^16^ Furthermore, 30%–50% of the population of Latin American counties rely on public healthcare, but funding for these services, which is derived from federal taxation, is inadequate. As a result, most public hospitals operate with very limited budgets and lack resources to establish ASPs or other programs and tools needed for optimal ASPs, such as robust microbiology laboratory capacity and implementation of electronic health records, from which antibiotic use data can be obtained.^17^ Most countries in Latin America do not have federal legislation or regulations that compel hospitals to implement ASPs or prioritize efforts to combat AMR. The World Health Organization (WHO) through its regional office, the Pan American Health Organization (PAHO), has been working to address some of these gaps, including development of policy guidance to facilitate implementation of national antimicrobial stewardship activities.^18^ The guidance includes, among other activities, regulation of remuneration policies to promote responsible antimicrobial prescribing.

### Behavioral determinants

Social and cultural factors can impact the effectiveness of ASPs in Latin America.^19^ Correlation between cultural determinants and both antimicrobial use and AMR have been described. At the country level, societal characteristics (eg, high power differentials, in which subordinates are not encouraged to speak up and participate in the decision-making process, and those in which individual goals are valued more than collaboration and teamwork) are associated with an increased prevalence of methicillin-resistant *Staphylococcus aureus* (MRSA) and extended-spectrum β-lactamase–producing organisms, antibiotic use, and inferior IPC practices compared with societies in which these characteristics do not predominate.^20,21^ A study of cultural determinants in Guatemala reported that only 53% of prescribers were highly receptive to recommendations from the antimicrobial stewardship team, and almost 40% felt that it was wrong to make modifications to antibiotics prescribed by a colleague (C.S., personal communication). These cultural determinants may represent critical barriers to integrating pharmacists, bedside nurses, and infection preventionists in antimicrobial stewardship and to empowering them to lead quality improvement interventions in Latin America.

Another important cultural determinant that can result in inappropriate antibiotic prescribing is uncertainty avoidance.^22^ Clinicians and patients historically have been reassured by prescribing and taking antibiotics, respectively. Understanding what may help mitigate uncertainty avoidance is crucial to designing strategies to improve prescribing habits. For example, while ambulatory prescribers in the United States indicated that being concerned about adverse events may help reduce inappropriate antibiotics^23^; this was not prioritized by ICU prescribers in England whose antibiotic perceptions were strongly influenced by beliefs that antibiotics would protect patients from deterioration and themselves from the ethical and legal consequences of undertreatment.^22^ These findings highlight the need to better understand prescribers’ perspectives on antibiotic management in different settings to develop effective approaches to improve antibiotic use in different patient populations. Recently, in the United States, Cosgrove et al^24^ led a national program to improve hospital antibiotic use built on patient safety. The program included training of frontline providers in technical (eg, medical knowledge) and adaptive factors (eg, patient safety, communication, teamwork). The authors found that hospitals more actively engaged in the program had greater reduction in antibiotic use than those with lower engagement, with −34.2 days of therapy versus −15.6 DOT, respectively, between the beginning and end of the program. A separate intervention that focused on education on patient safety, quality improvement, effective teamwork, and communication resulted in a 30% increase in compliance with infection prevention best practices and a significant reduction in central-line–associated bloodstream infections in ICUs from 5 countries in Latin America^25^; however, this approach has not been utilized to improve antibiotic prescribing in the region, although it may be key to changing behavior regarding antibiotic use.

### Contextual determinants

#### Infection prevention and control

Reduction in AMR depends on both decreasing the selective pressure exerted by antibiotics that can lead to emergence of resistance as well as strong IPC activities to prevent transmission of resistant organisms. MDR organisms are transmitted via hands of healthcare personnel, through contaminated medical equipment, or via an environmental reservoir (particularly *Pseudomonas* and *Acinetobacter*, which can persist in aqueous environments). Transmission of resistant organisms to patients must be addressed through robust infection prevention approaches, and hospitals must support and allocate resources for both antimicrobial stewardship and IPC activities in Latin America.^26,27^ This issue was nicely illustrated in the study by Lopardo et al,^28^ which showed an increase in use of colistin and carbapenems associated with an outbreak of *Acinetobacter* and *Pseudomonas* related to suboptimal IPC practices despite having an ASP in place with prospective review and feedback.

#### Treatment guidelines

According to the literature, 60%–80% of hospitals in Latin America report having infectious diseases treatment guidelines and 50%–60% have measured compliance^9,11,29^; however, data on whether these guidelines are based on international standards or adapted to local epidemiology are limited. Feinstein et al^13^ reported improved patient outcomes for patients presenting with complicated urinary tract infections when emergency room prescribers were compliant with ASP-developed guidelines that took into account risk factors for MDR bacteria and severity of illness in a hospital in Colombia with a high MDR prevalence. Guideline adaption to local epidemiology is particularly important for Latin America, where AMR differs significantly between countries and between regions within a country.^30^ Accomplishing this critical antimicrobial stewardship activity requires time and resources because it involves a multidisciplinary team.

#### Resources

**Fig. 1.**
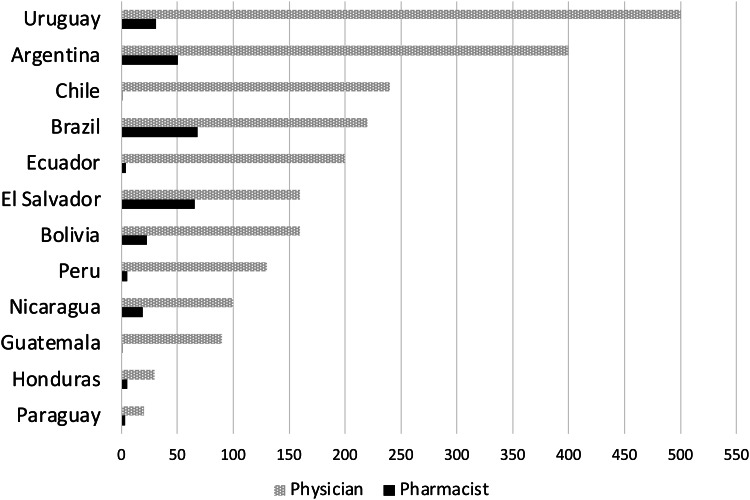
Physician and pharmacist per 100,000 people in Central and South American countries. Source: World Health Organization’s Global Health Workforce Statistics and Global Health Observatory.

Physicians and pharmacists have complementary skills and expertise in antimicrobial stewardship. An important barrier to ASP implementation in Latin America has been the lack of availability of physicians and clinical pharmacists. Figure 1 shows the physician and pharmacist-to-population ratio in several countries of Central and South America. Even when healthcare professionals are available, they may not have the training needed to perform antimicrobial stewardship activities. The role of Latin American pharmacists is still largely confined to drug distribution, or as members of the antimicrobial stewardship or IPC committees without an active role in making recommendations about antibiotic selection or dosing. Several examples of successful models support a leadership role by pharmacists in ASPs, such as remote support by an expert antimicrobial stewardship team and train-the-trainer programs.^31^ Some countries in Latin America and the Caribbean (eg, Barbados, Brazil, Costa Rica, and Colombia) have already implemented standards or have passed laws regarding the role of clinical pharmacists in hospital ASPs; however, this remains largely unaddressed in most countries in the region. In addition to pharmacists, other healthcare professionals can contribute to antibiotic stewardship efforts. Recently, the role of bedside nurses in antimicrobial stewardship has been emphasized. Nurses can help optimize use of microbiologic tests, such as avoiding unnecessary urine or sputum cultures (which often result in unnecessary antibiotic treatment) or collecting specimens appropriately for accurate results, including aseptic techniques and specimen collection prior to antibiotic administration.^32^ Another area in which nurses can contribute to antimicrobial stewardship efforts is appropriate documentation of antibiotic allergies.

Infection preventionists are expected to work closely with antimicrobial stewardship colleagues, and in some countries, they have been tasked with implementing antimicrobial stewardship activities as well. In these situations, careful balance of activities and allocation of resources and support of individuals with drug expertise and knowledge on infectious diseases management must be considered to ensure appropriate dedication to both activities. Recommendations on the minimum requirement for successful antimicrobial stewardship and IPC programs have been published.^33,34^ The minimum recommended full-time equivalent support (includes physician and pharmacist) for a successful ASP is 1 for a 100-bed hospital, 1.3 for a 101–300-bed hospital, 1.6 for a 301–500-bed hospital, 2.6 for a 501–1,000-bed hospital, and 4 for a >1,000-bed hospital.

Improvement is needed in hospital access to technology both to track antibiotic use and AMR more efficiently and to help display these data in a meaningful way to the ASP for action, as well as improving clinicians’ access to evidence-based information (eg, treatment guidelines, dose recommendations) at the point of care, which can be challenging in Latin America where computer availability and access to scientific journals are limited. To address these needs, a free phone app and a free web platform have been developed and are available in all countries.^11,35^ This free platform allows for longitudinal storage of deidentified data such as antibiotic consumption, point-prevalence survey data, and ASP core elements data.^11^ Hospitals can track their own data over time and compare themselves against other hospitals in the same country (benchmarking). Studies to evaluate the impact of the platform are needed. The app contains treatment guidelines that hospitals can adapt based on their local epidemiology antimicrobial-specific information (eg, dosing, side effects, drug interactions) and renal- and liver-function calculators.

Finally, increased microbiology capacity is needed for accurate detection of resistance and to improve access to results at the point of care for timely antimicrobial actions. This barrier is more challenging to overcome because digitalization of electronic medical records is costly both to implement and maintain. Free resources for antimicrobial stewardship implementation are summarized in the Supplementary Table.

## Addressing antimicrobial stewardship gaps in Latin America

### Evaluation of program structure and interventions

AMR remains a public health emergency in Latin America.^36^ National action plans have slowly been developed and implemented, and hospitals antimicrobial stewardship activities have grown despite limited allocated resources. In addition to better resource allocation, there needs to be an evaluation of how current hospital reimbursement models affect implementation of effective ASPs.^37^

Previous assessments of ASPs have focused on traditional evaluations of program structure, processes, and outcomes. Although these are fundamental aspects to evaluate, assessments should be complemented with evaluations that incorporate behavior and context determinants, which occur at the individual level as well as the wider organizational and social levels and can affect the implementation of antimicrobial stewardship activities.

**Fig. 2.**
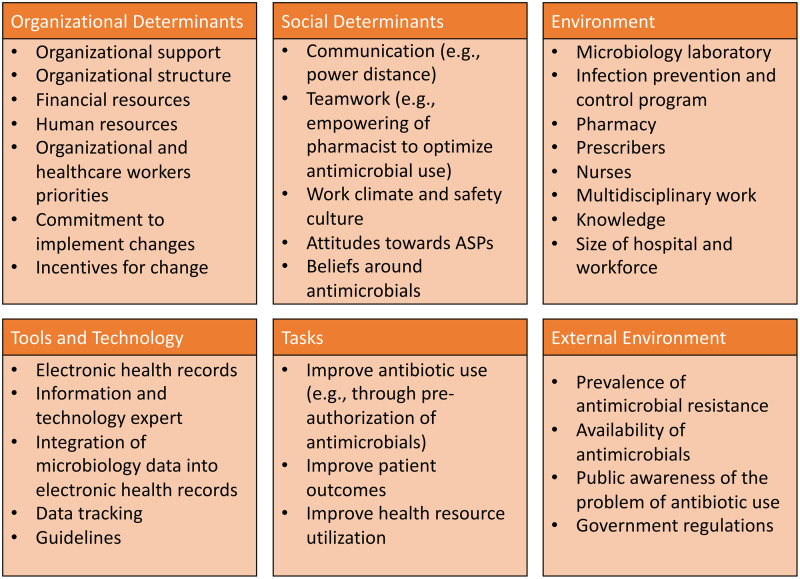
Suggested domains and determinants to consider for successful implementation of antimicrobial stewardship activities.

Several determinant frameworks include a wide range of factors (determinants) that may influence outcomes and program implementation and that should be considered to decrease the risk of implementation failures.^38^ These frameworks are used to assess potential barriers and facilitators within local settings (examples of frameworks that have been applied to antimicrobial stewardship are summarized in Table 1). Determinant frameworks have been applied to improve antibiotic use in ambulatory settings,^39,40^ but they have not been explored as thoroughly for inpatient antimicrobial stewardship activities. For example, while a traditional evaluation of an ASP would inquire about access to the microbiology laboratory and the availability of rapid microbiologic diagnostic testing to inform antibiotic decisions, a determinants framework-based evaluation would help obtain more granular data regarding how successfully the microbiology laboratory has been integrated into antimicrobial stewardship by taking into account the microbiology personnel (eg, training of staff to perform newer diagnostic technologies, human resources to staff the lab, lab hours of operation), mechanisms to provide timely results to prescribers, and prescribers perceptions and attitudes towards rapid diagnostics (eg, whether prescribers are acting upon results). A summary of domains and potential determinants to consider when evaluating antimicrobial stewardship interventions in Latin America is presented in Figure 2. Lastly, better understanding of factors associated with successful ASPs in Latin America is needed to promote antimicrobial stewardship success in the region.

**Table 1.** Determinant Frameworks That Have Been Used to Implement Antibiotic Stewardship

Framework	Origin	Focus	Characteristics
Systems Engineering Initiative in Patient Safety (SEIPS)	Human-factors engineering model developed specifically for healthcare settings	Diagnosis of gaps and barriers Focuses on supporting individuals through the work system to improve outcomes	1. Workplace (eg, inpatient unit) 2. Work system (includes individuals, tools and technologies, organization, tasks, physical environment, larger external environment) 3. Process (antibiotic prescribing) 4. Outcomes (antimicrobial resistance)
Consolidated Framework for Implementation Research (CFIR)	Organizational change and psychology	Diagnosis of gaps and barriers with emphasis on multilevel factors	1. Characteristics of the intervention (complexity of intervention, evidence for the intervention, adaptation to local needs) 2. Outer setting (patient needs, network with other hospitals, external policies, and incentives) 3. Inner setting (organization characteristics, culture, readiness for change, priorities, resources) 4. Characteristics of individuals (knowledge, beliefs) 5. Process (plan, engage, leadership support, champions, execute intervention, evaluate progress)
Capability, Opportunity, and Motivation for Behavior change (COM-B)	Model used to increase vaccination uptake among populations with suboptimal vaccine coverage	TAP tool kit helps making a diagnosis of gaps and with the process of implementation (ie, engage, analyze, prioritize, design the intervention, implement, and evaluate) Focuses on drivers and barriers to behaviors (antibiotic prescription) and is included in the Tailoring Antimicrobial Resistance Programs (TAP) tool kit	1. Capability • Prescriber and patient knowledge of the problem of inappropriate antibiotic use • Prescriber knowledge on drivers of AMR • Belief prescriber and patient can influence AMR • Skills to communicate effectively about antibiotics 2. Opportunity • Access to antibiotic data, guides, micro results • Regulations • Culture, social norms, and values 3. Motivation • Beliefs on rational antibiotic use • Prescriber values regarding patient safety and prescribing habits • Factors that may influence antibiotic decision making

### Improving training in antimicrobial stewardship

As previously discussed, increased ID and antimicrobial stewardship expertise is needed among the healthcare work force, including the members of the antimicrobial stewardship team. Several regular training courses exist; although many are in English and could be quite expensive, which challenge wider use in Latin America. Recently, free training programs on antimicrobial stewardship implementation have become available online in Spanish^41^; however, more training on strategic planning and systematic approaches to quality improvement (eg, plan–do–study–act model), as well as other key factors, are needed for successful ASPs, such as guideline development, how to approach the C-suite, and how to adopt and promote teamwork and effective communication.

As previously mentioned, better promotion of safety culture and better training of healthcare workers are needed to empower pharmacists to take a more active role in antimicrobial stewardship and to help prescribers accept their recommendations.

### Evaluation of antimicrobial use

Another area for improvement in Latin America is quantifying and benchmarking antibiotic use. Point-prevalence surveys of hospital antibiotic use have provided useful data to define antimicrobial stewardship priorities.^11,27,42^ These include the most common infectious syndromes and prescribed antibiotics, and certain practices such as lack of microbiologic cultures prior to initiating antimicrobial treatment,^27^ and the high use of certain antibiotics despite high prevalence of organisms being resistant to these antibiotics.^42^

The limitations of the point-prevalence survey methodology include misclassification of patients due to bed shortages, discordant data when mixed electronic and paper records exist, and lack of familiarity among data abstractors with electronic health records. These issues are exacerbated in hospitals in resource-limited settings.^43^ Point-prevalence surveys do not allow for evaluation of duration of therapy, and more research is needed to understand current antibiotic prescribing patterns with respect to duration because this has been a major driver of inappropriate antibiotic use in other countries such as the United States. As a start, assessing prescriber’s awareness on duration of therapy by evaluating documentation of duration in the chart during point-prevalence surveys could shed some light on this topic. Antibiotics are used among higher proportions of ICU patients relative ward patients in Latin America. Hence, given limited resources, most antimicrobial stewardship activities in Latin American hospitals have concentrated on the ICU. However, most hospitalized patients are outside the ICU, and better understanding of antibiotic use is needed to improve the reach of antimicrobial stewardship to non-ICU patients.^9,42^

In summary, antimicrobial stewardship resources and antimicrobial stewardship activities differ significantly among Latin American countries. Progress has been made in antimicrobial stewardship across Latin America at the hospital level, but it has been slow. Better understanding of the major challenges is needed to promote effective antimicrobial stewardship in the region, including through evaluation of the culture of safety and human factors that influence antibiotic prescribing.

## Supplementary material

For supplementary material accompanying this paper visit https://doi.org/10.1017/ash.2022.47. click here to view supplementary material
