# Supplementary material for: Antimicrobial stewardship in Latin America: Past, present, and future
Source: Antimicrob Steward Healthc Epidemiol. 2022 Apr 22;2(1):e68. doi: 10.1017/ash.2022.47 (PMC9726506; doi:10.1017/ash.2022.47)
Supplement: Supplementary file 1 [file S2732494X2200047Xsup001.docx]

**Supplementary Table:** Free resources for Antimicrobial Stewardship (AS) Program implementation

| **Item** | **Language** | **Where to find it** | **Notes** |
| --- | --- | --- | --- |
| **Program self-assessment tools** | | | |
| - Antimicrobial stewardship programmes in health-care facilities in low- and middle-income countries: a WHO practical toolkit | - Spanish | - <https://apps.who.int/iris/bitstream/handle/10665/335947/9789240003057-spa.pdf?sequence=1&isAllowed=y> | - Comprehensive guide for implementation of AS programs in low- and middle-income countries. - Includes:   - How to perform a gap analysis   - Approach to antibiotics (AWaRe classification)   - How to implement AS interventions   - How to assess the impact of the AS program   - Contains indicators with questions for “basic” and “advanced” core elements |
|  | - English | - <https://apps.who.int/iris/handle/10665/329404> |  |
| - WHO policy guidance on integrated antimicrobial stewardship activities | - Spanish | - <https://www.who.int/es/publications/i/item/9789240025530> | - Guide for implementation of AS activities at the national and facility level. - Contains self-assessment tools for the national and hospital levels (assesses core elements such as leadership support, AS actions, surveillance, etc.). |
|  | - English | - <https://www.who.int/publications/i/item/9789240025530> |  |
| - ASP in Adult Intensive Care Units in Latin America | - English/Spanish (upon request to first author) | - Supplementary material in Quirós RE et al; PROA-LATAM Project Group. Infect Control Hosp Epidemiol. 2022, PMID  33829982 | - This self-assessment tool was adapted from the CDC core elements to the Latin America region. - Provides a score (0-100) - Validated with data on antibiotic use and multidrug resistance organisms (i.e., higher score correlated with better antibiotic use, less antibiotic consumption, and lower incidence of multidrug resistant organisms) |
| **Educational resources for the ASP** | | | |
| - Agency for Healthcare Research and Quality Safety Program for Improving Antibiotic Use | - English | - Acute care hospital Toolkit:   <https://www.ahrq.gov/hai/tools/antibiotic-stewardship/index.html>   - Long term care Toolkit:   <https://www.ahrq.gov/antibiotic-use/long-term-care/index.html> | - National program developed to expand ASP implementation in the United States. - Goal is to foster antibiotic stewardship as a patient safety event. - Directed to those leading AS activities. - PowerPoint slides with facilitator guides. - Covers:   - How to develop a culture of safety around antibiotic prescribing   - How to develop and improve an ASP   - Applying the 4 moments framework for antibiotic prescribing   - Best practices for the diagnosis and treatment of infectious diseases |
| - Toolkit to Enhance Nursing and Antibiotic Stewardship Partnership | - English | - Available at:   <https://www.hopkinsmedicine.org/antimicrobial-stewardship/nursing-toolkit/index.html> | - Toolkit to implement nurse-driven antibiotic stewardship activities. - Includes:   - Implementation checklist and framework   - Educational modules for nurses (Urinary tract infections and urine cultures, pneumonia, and respiratory cultures, *Clostridioides difficile* testing, documentation of penicillin allergy)   - Algorithms to optimize urine and respiratory cultures   - Algorithms to optimize allergy history |
| - Curso para la implementación y el fortalecimiento de los Programas de Optimización de Antimicrobianos (PROA) | - Spanish | - Available at:   <https://www.campusvirtualsp.org/es/curso/curso-para-la-implementacion-y-el-fortalecimiento-de-los-programas-de-optimizacion-de> | - Periodic training by PAHO (usually every year) - Five modules over 7 weeks |
| **ASP technological resources** | | | |
| *Data tracking* | | | |
| - PROAnet | - Spanish | - Available at:   [www.proanet.org](http://www.proanet.org) | - Free secured platform available for all countries. - One-step registration process - User-friendly, provides an efficient way to visualize data and track it over time - Structured to house:   - Self-assessment   - Point prevalence survey data for antimicrobial use   - Antimicrobial consumption data   - Incidence of multidrug resistance organisms   - Crude mortality   - Allows tracking data over time   - Allows benchmarking to other hospitals in the country |
| *Clinical decision support tools* | | | |
| - PROAnet app | - Spanish | - Available in Google play and App Store | - Free app - Contains:   - Clinical guidelines that can be adapted to local epidemiology   - Antimicrobial information (e.g., dosing, drug interactions, safety in pregnancy, side effects)   - Renal and liver function calculator   - Allows uploading of local antibiograms |
| **General information on antimicrobial resistance per country** | | | |
| - Latin American Network for Antimicrobial Resistance Surveillance (Pan American Health Organization established network) | - Spanish | - Available at:   <https://www.paho.org/es/temas/resistencia-antimicrobianos/red-latinoamericana-caribe-vigilancia-resistencia-antimicrobianos>  and at:  https://www3.paho.org/data/index.php/es/temas/resistencia-antimicrobiana.html | - One of the largest regional AMR surveillance networks in the world (over 750 laboratories within the region have reported AST data on a total of ~2,633,000 isolates). - It includes data from 20 countries. Aggregates level standardized data on antibiotic resistance from both community and nosocomial pathogens is collected and reported annually, on number of priority pathogens-drugs combination. |
|  | - English | - Available at:   <https://www.paho.org/en/topics/antimicrobial-resistance/latin-american-network-antimicrobial-resistance-surveillance>  and at:  https://www3.paho.org/data/index.php/en/mnu-topics/antimicrobial-resistance.html |  |
| **Other implementation guides** | | | |
| - Proposed roadmap for the adaptation and implementation of WHO policy guidance on integrated antimicrobial stewardship in the human health sector | - Spanish | - <https://www.paho.org/es/documentos/propuesta-hoja-ruta-para-adaptacion-e-implementacion-politica-oms-sobre-actividades>) | - This document provides a roadmap for implementation of the WHO policy on integrated activities to optimize antimicrobial use in human health - The roadmap includes using existing resources, gap analysis, multidisciplinary work, establishing goals and assessing needed resources |
|  | - English | - <https://www.paho.org/en/documents/proposed-roadmap-adaptation-and-implementation-who-policy-guidance-integrated> |  |
| - Core Elements of Human Antibiotic Stewardship Programs in Resource-Limited Settings | - English | - <https://www.cdc.gov/antibiotic-use/healthcare/pdfs/stewardship-resource-limited-508.pdf> | - Based on the CDC Core elements, this guide outlines “basic” and “advanced” AS activities for implementation based on local resources |
| - Recommendations for Implementing Antimicrobial Stewardship Programs in Latin America and the Caribbean: Manual for Public Health Decision-Makers | - English | - https://iris.paho.org/handle/10665.2/49645 | - This manual examines the concept and benefits of AS programs, and describes their major components: leadership, human resources, microbiology laboratories, and robust pharmaceutical services. - Also includes AS strategies in the ambulatory setting, including legislation around over-the-counter medications. |
